# Robotic distal pancreatectomy with or without preservation of spleen: a technical note

**DOI:** 10.1186/1477-7819-12-295

**Published:** 2014-09-23

**Authors:** Amilcare Parisi, Francesco Coratti, Roberto Cirocchi, Veronica Grassi, Jacopo Desiderio, Federico Farinacci, Francesco Ricci, Olga Adamenko, Anastasia Iliana Economou, Alban Cacurri, Stefano Trastulli, Claudio Renzi, Elisa Castellani, Giorgio Di Rocco, Adriano Redler, Alberto Santoro, Andrea Coratti

**Affiliations:** Department of Digestive and Liver Surgery Unit, St Maria Hospital, Viale Tristano di Joannuccio 1, 05100 Terni, Italy; Department of General Surgery, Misericordia Hospital, Via Senese 169, 58100 Grosseto, Italy; Department of General and Oncologic Surgery, University of Perugia, St. Maria Hospital, Località Sant’Andrea delle Fratte, Piazzale Menghini 1, 06156 Perugia, Italy; Department of Surgical Sciences, ‘Sapienza’ University of Rome, Viale Regina Elena 324, 00185 Rome, Italy

**Keywords:** pancreatic surgery, robotic surgery

## Abstract

**Background:**

Distal pancreatectomy (DP) is a surgical procedure performed to remove the pancreatic tail jointly with a variable part of the pancreatic body and including a spleen resection in the case of conventional distal pancreatectomy or not in the spleen-preserving distal pancreatectomy.

**Methods:**

In this article, we describe a standardized operative technique for fully robotic distal pancreatectomy.

**Results:**

In the last decade, the use of robotic systems has become increasingly common as an approach for benign and malignant pancreatic disease treatment. Robotic Distal Pancreatectomy (RDP) is an emerging technology for which sufficient data to draw definitive conclusions in surgical oncology are still not available because the follow-up period after surgery is too short (less than 2 years).

**Conclusions:**

RDP is an emerging technology for which sufficient data to draw definitive conclusions of value in surgical oncology are still not available, however this techniques is safe and reproducible by surgeons that possess adequate skills.

## Background

Distal pancreatectomy (DP) is a surgical procedure performed to remove the pancreatic tail jointly with a variable part of the pancreatic body and including a spleen resection in the case of conventional distal pancreatectomy or not in the spleen-preserving distal pancreatectomy. In this article we describe a technical note on RDP.

## Methods

### Operative technique

**Figure 1:**
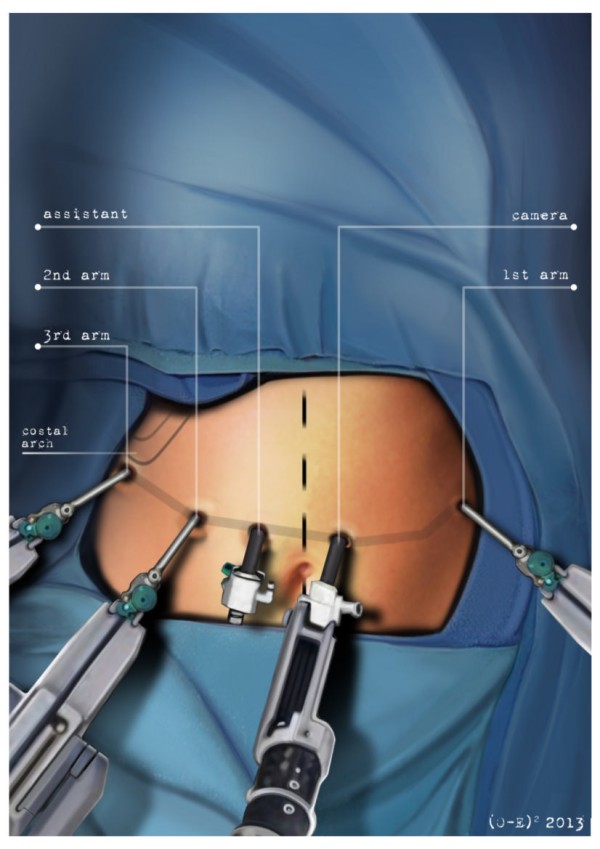
**The trocars are placed following a concave and arcuated line.**

**Figure 2:**
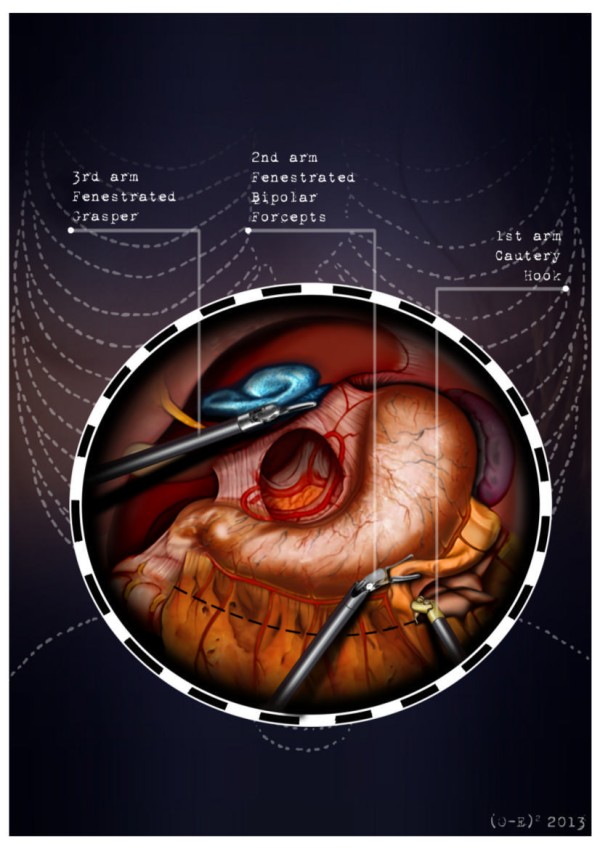
**The gastrocolic ligament is cut from the right to the left side with the help of a cautery hook, until complete exposure of the pancreatic isthmus is obtained and the gastrolienal ligament is reached.**

**Figure 3:**
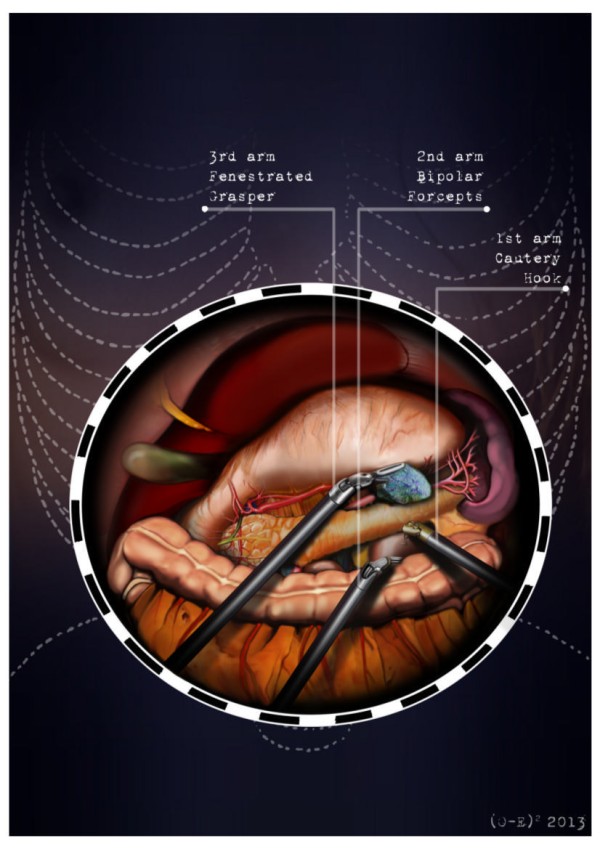
**The stomach is lifted upward by the third robotic arm and the transverse colon is moved downwards.**

**Figure 4:**
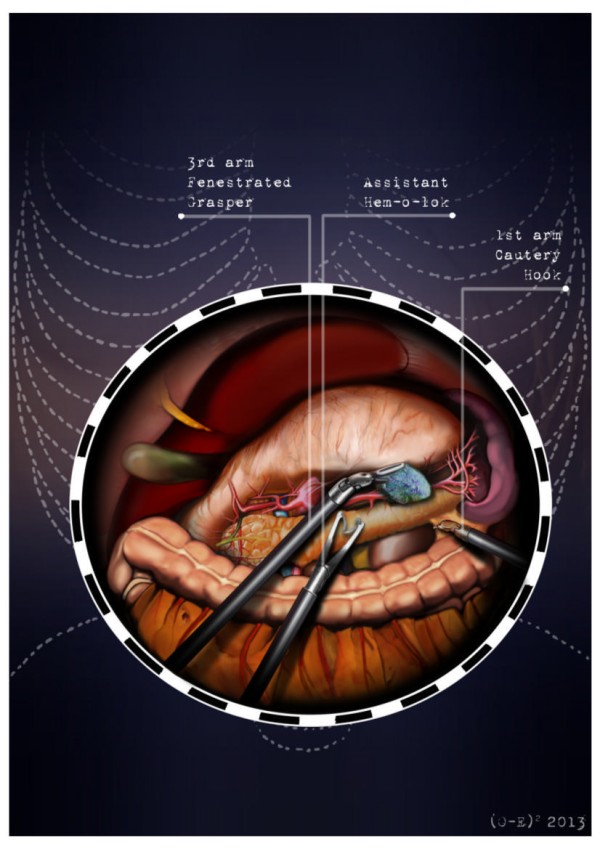
**Splenic artery dissection.**

**Figure 5:**
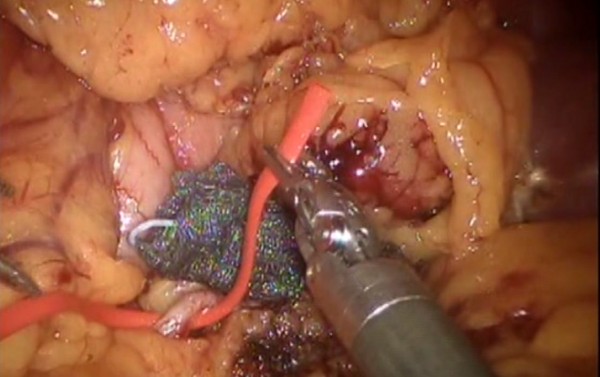
**Splenic artery ligature with Hem-o-loks and sectioning.**

**Figure 6:**
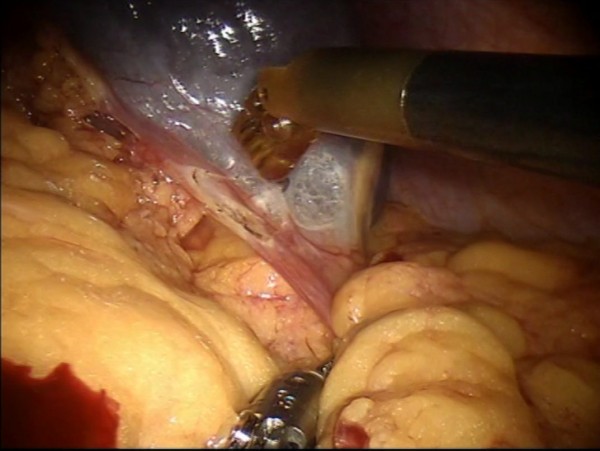
**Section of the splenorenal ligament by the cautery hook.**

**Figure 7:**
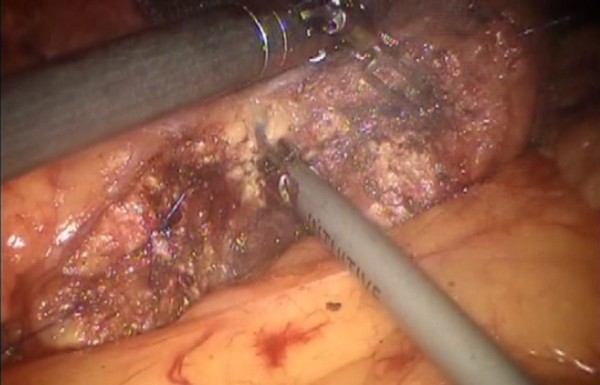
**Pancreatic section.**

**Figure 8:**
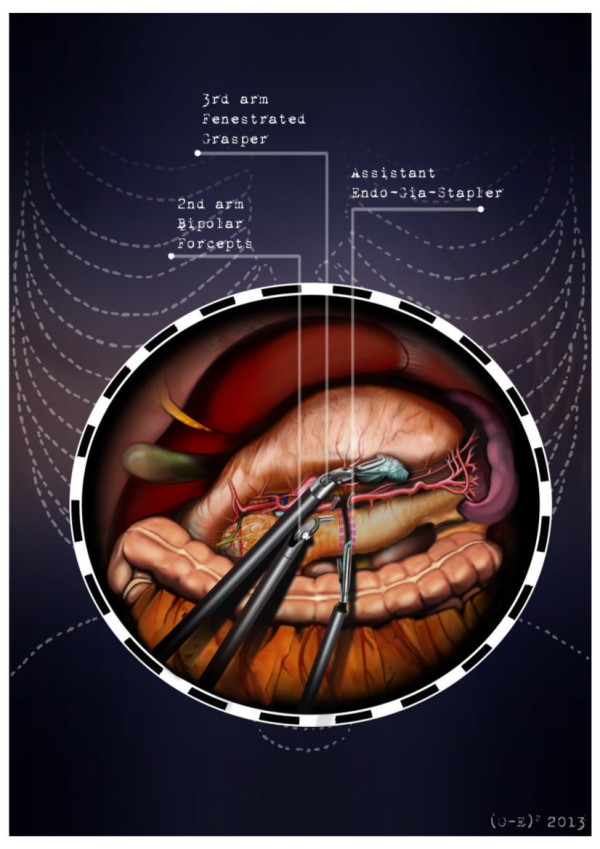
**The pancreatic section is performed with robotic Ultracision, placed on the arm no 1, gradually reaching Wirsung’s duct.**

**Figure 9:**
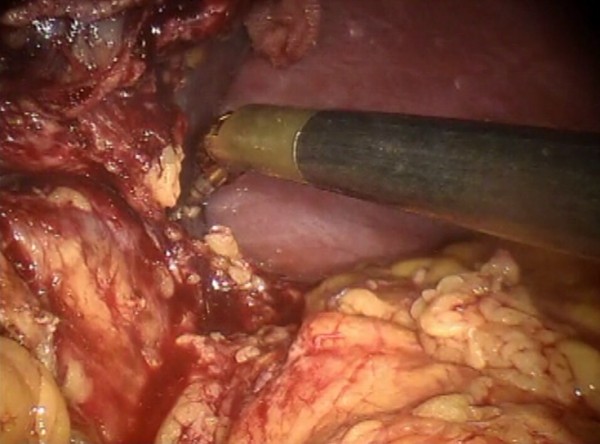
**Pancreas is isolated from the posterior abdominal wall by dissecting along the soft avascular tissue behind the retropancreatic band and the splenic ilum.**

**Figure 10:**
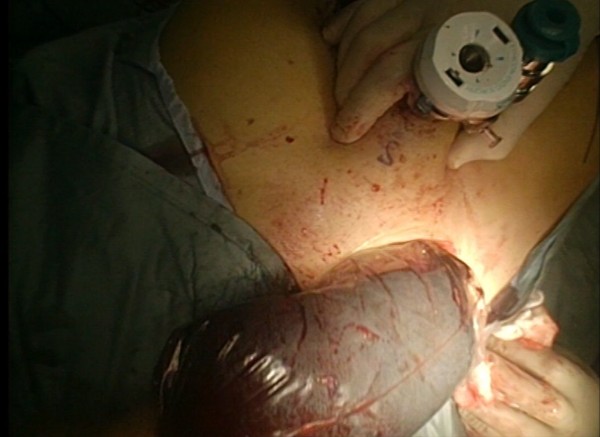
**Surgical specimen is extracted with an Endocath through a McBurney or Pfannenstiel abdominal incision.**

After the induction of general anesthesia, the patient’s arms are abducted and his legs are spread apart in order to allow the placement of the assistant surgeon. A nasogastric tube and urinary catheter are also applied. After preparation of the skin with povidone-iodine is completed, the abdomen is insufflated with CO2 using a veress needle through a one millimeter diameter periumbilical incision. The ınsufflator is set to a constant pressure of 12 mmHg. The trocars are placed following a concave and arcuate line (Figure 1). Usually, the optical trocar is inserted just above and to the left of the umbilicus. In practice, however, its position could vary in relation to the patient’s anatomy and pancreatic lesion localization, which is why a preliminary introduction of an assistant 12-mm extra port on the transverse umbilical line in between the xifopubic and left middle axillary line could be useful in order to check the internal anatomy and evaluate the optimal position of the optical trocar. The first robotic trocar is positioned at the intersection of the left middle axillary line and the transverse umbilical line, the second robotic trocar at the intersection of the right anterior axillary line and the transverse umbilical line, and the third robotic trocar in the right hypochondrium. The assistant surgeon in the various surgical phases will be able to introduce an aspirator, a pair of forceps, a mechanical stapler or a suture thread through the assistant port. The robotic cart is placed between the patient’s head and left shoulder after rotating the operation table to the right and consequently docking the robotic system. The robotic camera is inserted through the periumbilical trocar port, the cautery hook is placed on arm number 1, the fenestrated bipolar forceps is placed on arm number 2, and the double fenestrated grasper on arm number 3. The gastrocolic ligament is cut from the right to the left side with the help of a cautery hook, until complete exposure of the pancreatic isthmus is obtained and the gastrolienal ligament is reached (Figure 2). Subsequently, the short gastric vessels are meticulously identified and dissected by ultrasound dissector or bipolar forceps; when necessary clips and Hem-o-loks could also be applied. The stomach is lifted upward by the third robotic arm, and the transverse colon is moved downwards (Figure 3). In this manner a passage that leads to the lesser sac is obtained, helping us to distinguish and dissect the splenic artery at the superior pancreatic edge. The artery is ligated distally using Hem-o-loks and sectioned (Figures 4 and 5). The colosplenic ligament is sectioned so that the spleen is completely mobilized. The inferior spleen pole is pulled to the right with the help of a pair of fenestrated bipolar forceps, thus allowing the complete section of the splenorenal ligament by the cautery hook (Figure 6). During this procedure, attention must be paid to avoid injury to the left adrenal gland. This moment is particularly important as it identifies the precise level for the forthcoming dissection. Dissection of the lower edge of the pancreas should be performed following a retropancreatic avascular plane of dissection until visualization of the splenic vein on the posterior surface of the gland. Before ligature, the splenic vein should be isolated from the fibrotic lamina surrounding it. The splenic vein could be sectioned using proximal and distal ligatures with a Hem-o-lok or stapler. Two suspension sutures are placed at the lower edge of the pancreas at the expected level of gland resection. The pancreatic section is performed with robotic Ultracision, placed on the arm number 1, gradually reaching the duct of Wirsung, which must be tied before it is sectioned (Figures 7 and 8). Alternatively, this step can be performed using a mechanical stapler. The pancreas is finally isolated from the posterior abdominal wall by dissecting along the soft avascular tissue behind the retropancreatic band and the splenic hilum, until complete mobilization of both the organs (Figure 9). After checking the correct detachment of the surgical specimen, it is extracted with an Endocath through a McBurney or Pfannenstiel abdominal incision (Figure 10). After checking the hemostasis, a Jackson-Pratt drain is placed close to the site of the pancreatic section and incisions are sutured.

## Results and discussion

In 1913, Mayo standardized the surgical procedure for DP [1], after the first described DP was performed by Trendelemburg in a case of pancreatic sarcoma [2]. Currently, there are reports that describe safely performing a spleen preserving pancreatectomy in cases of trauma, benign lesions of the body and tail of the pancreas next to the duct of Wirsung, or chronic pancreatitis. Spleen preservation allows many well-demonstrated advantages in terms of morbidity and mortality, preventing the development of infections and facilitating a faster postoperative recovery [3]. However this type of surgical intervention is rarely performed due to the need to select patients, technical difficulties, and the dependence of these procedures on the experience of the surgeon. Mallet-Guy standardized the technique of DP with spleen preservation in chronic pancreatitis: the splenic vessels are identified and dissected from the posterior portion of the gland, followed by the resection of the body/tail of the pancreas [4]. Quenu and Leger point out a collateral blood circulation that can be used to preserve the spleen through the short gastric vessels and the gastroepiploic vessels. Their technique may also be used in the case of interruption of the blood flow of the splenic vessels caused by their iatrogenic rupture or section. Some authors, Leger among others, underline the risk of developing a segmental portal hypertension and suggest performing splenectomy when it is not possible to preserve the splenic vein [5]. In 1988, Warshaw revised the spleen-preserving DP and showed that the use of the short gastric vessels is not only useful to preserve the spleen in the case of damage to the splenic vessels but can also be exploited as a technique of choice in selected cases [6, 7]. The advent of laparoscopy has led to evaluation of the feasibility of a minimally invasive approach for DP. In 1994 Cuschieri performed the first laparoscopic distal pancreatectomy (LDP) [8], followed by Gagner *et al*., who presented their experience on this topic [9]. Thereafter, a large number of studies reported results; nevertheless, all of them are limited by a small sample size [10–13]. LDP is a procedure considered technically demanding due to the known limitations of the traditional laparoscopic approach. In the last decade, the use of robotic systems has become increasingly common as an approach for benign and malignant pancreatic disease treatment. The robotic system adds precision to the movements and greatly increases the comfort of the surgeon dealing with a delicate minimally invasive dissection phase. Robotic surgical system instrumentation allows the use of a magnified and three-dimensional viewing field [14, 15], a steady traction, tremor suppression [16], flexibility of the instruments [17], and thus, safe suturing. A recent literature review of robotic distal pancreatectomy (RDP) shows that RDP is an emergent technology, for which there is, as yet, insufficient data to draw definitive benefit with respect to conventional or laparoscopic surgery. The mean duration of RDP is longer with the Da Vinci robot, but the hospital stay is shorter even if influenced by different hospital protocols [18]. However, we cannot reach a precise conclusion on the indications for the different approaches because the number of patients treated with the robot is low, studies presented in the literature present a small number of patients, and randomized trials are absent. In this article we describe a technical note on RDP.

## Conclusions

RDP is an emerging technology for which sufficient data to draw definitive conclusions of value in surgical oncology are still not available and for which the follow-up period after surgery is too short (less than 2 years) [18]; however this techniques is safe and reproducible by experienced surgeons. We performed an update of the literature review from January 2003 to February 2014; we found 31 studies, whose characteristics are reported in Table 1. None of the studies was a randomized clinical trial. The definition of the robotic approach was heterogeneous: the technique was defined as fully robotic, robotic, robotic-assisted, robot-assisted laparoscopic and hybrid robotic [19–47]. The dissection and resection were also heterogeneous, sequentially combining different approaches: laparoscopic/robotic and only robotic. In this article we have presented a standardized operative technique for fully robotic distal pancreatectomy.

**Table 1:** **Review of the literature**

Study (Author/year/type)	Duration (year)	Setting City Nation	Patients	Author’s definition of Robotic DP	Type of dissection and resection
**Han [** [19]**] 2014 Case report**	2013	Seoul South Korea	1	Robotic RAMPS	Robotic
**Hanna [** [20]**] 2013 CCT**	2006-2012	Charlotte, NC, USA	39	Robotic-assisted laparoscopic distal pancreatectomy	Robotic-laparoscopic
**Zhang [** [21]**] 2013 Review**		Beijing, China		Robotic-assisted distal pancreatectomy	
**Milone [** [22]**] 2013 Review**		Chicago, IL, USA		Robotic distal pancreatectomy	
**Benizri [** [23]**] 2013 CCT**	2004-2011	Vandoeuvre-les-Nancy, France	11	Robot-assisted distal pancreatectomy	Robotic
**Fernandes [** [24]**] 2013 Review**		Chicago, IL, USA		RADP	Robotic
**Chen [** [25]**] 2013 Review**		Shanghai China		Robot-assisted distal pancreatectomy	
**Lai [** [26]**] 2013 Review**	2013	Hong Kong China		Robot-assisted laparoscopic distal pancreatectomy	
**Wayne [** [27]**] 2013 Case series**	2011-2012	New York, NY, USA	12	Robotic pancreatic distal resection	NR
**Jung** **[** [28]**]** **2013 Review**		Geneva, Switzerland		Robotic distal pancreatectomy	
**Strijker** **[** [29]**]** **2012 Review**		Utrecht Netherlands		Robot-assisted distal pancreatectomy distal pancreatectomy	
**Winer** **[** [30]**]** **2012 Review**		Pittsburgh, PA, USA		Minimally Invasive RADP	Robotic-laparoscopic
**Hwang** **[** [31]**]** **2012 CCT**	2007- 2011	Seoul South Korea	22	Robot-assisted spleen-preserving DP	Robotic
**Daouadi** **[** [32]**]** **2012 CCT**	2004- 2011	Pittsburgh, PA, USA	30	Minimally Invasive RADP	Robotic- laparoscopic
**Suman** **[** [33]**]** **2012 CCT**	2006- 2010	Ridgewood, NJ, USA	40	Robot spleen-preserving DP	NR
**Buturrini** **[** [34]**]** **2012 CCT**	NR	Verona Italy	5	Hybrid Robotic DP	Robotic-laparoscopic
				Fully Robotic DP	Robotic
**Choi** **[** [35]**]** **2012 Case series**	NR	Seoul South Korea	4	Robotic RAMPS	Robotic
**Kang** **[** [36]**]** **2011 CCT**	2006- 2010	Seoul South Korea	20	RADP	NR
**Ntourakis [** [37] **2011 Case report**	2010	Strasbourg France	1	Robotic Left Pancreatectomy	Robotic
**Chan** **[** [38]**]** **2011 Case series**	2009- 2010	Hong Kong China	2	Robotic spleen preserving DP	Robotic
**Kim** **[** [39]**]** **2011 Case report**	2009	Seoul South Korea	1	Robot Assisted spleen-preserving laparoscopic DP	Robotic
**Yiengpruksawan [** [40] **2011 Technical note**	2010	Ridgewood, NJ, USA	NR	RADP	Robotic-laparoscopic
**Ntourakis [** [41] **2010 Case series**	NR	Strasbourg France	2	Robotic Distal Splenopancreatectomy	Robotic
**Waters** **[** [42]**]** **2010 CCT**	2008- 2009	Indianapolis, IN, USA	17	Robotic DP	Robotic
**Giulianotti [** [43] **2010 Case series**	2000- 2007	Chicago, IL, and Grosseto, Italy	46	RADP	Robotic
**Vasilescu [** [44] **2009 Case report**	2008	Bucharest Romania	1	Robotic spleen-preserving DP	Robotic
**Machado [** [45] **2009 Case report**	NR	Sao Paulo Brazil	1	Robotic resection	Robotic-laparoscopic
**D’Annibale [** [46] **2006 Case series**	2001- 2004	Padova Italy	2	Robotic resection	Robotic
**Melvin** **[** [47]**]** **2003 Case report**	NR	Ohio OH, USA	1	Robotic resection	Robotic

**DP,** distal pancreatectomy; NR, not reported; **RADP,** robot-assisted distal pancreatectomy; **Robotic RAMPS,** robotic radical antegrade modular pancreatico-splenectomy.

## Consent

Written informed consent was obtained from the patient for the publication of this report and any accompanying images.

## Abbreviations

DP: distal pancreatectomy
LDP: laparoscopic distal pancreatectomy
NR: not reported
RADP: robot-assisted distal pancreatectomy
RAMPS: radical antegrade modular pancreatico-splenectomy
RDP: robotic distal pancreatectomy.
